# Population estimates, consequences, and risk factors of obesity among pregnant and postpartum women in India: Results from a national survey and policy recommendations

**DOI:** 10.1002/ijgo.13319

**Published:** 2020-09-07

**Authors:** Mansi Chopra, Naman Kaur, Konsam Dinachandra Singh, Chandni Maria Jacob, Hema Divakar, Giridhara R. Babu, Phuong Hong Nguyen, Arti Bhanot, Manisha Sabharwal, Sila Deb, Dinesh Baswal, Sarah Louise Killeen, Fionnuala M. McAuliffe, Mark A. Hanson, Vani Sethi

**Affiliations:** ^1^ National Centre of Excellence and Advanced Research on Diets Department of Food and Nutrition Lady Irwin College University of Delhi New Delhi India; ^2^ Institute of Developmental Sciences University of Southampton Southampton UK; ^3^ NIHR Southampton Biomedical Research Centre University Hospital Southampton Southampton UK; ^4^ Divakars Specialty Hospital Bengaluru India; ^5^ Department of Epidemiology Indian Institute of Public Health Public Health Foundation of India Bengaluru India; ^6^ International Food Policy Research Institute New Delhi India; ^7^ Ministry of Health and Family Welfare New Delhi India; ^8^ UCD Perinatal Research Centre School of Medicine National Maternity Hospital University College Dublin Dublin Ireland; ^9^ Nutrition Section United Nations Children’s Fund New Delhi India

**Keywords:** India, Obesity, Policy, Postpartum, Pregnancy, Prevalence, Risk factors of obesity

## Abstract

**Objective:**

To examine prevalence, risk factors, and consequences of maternal obesity; and provide evidence on current policies and programs to manage maternal obesity in India.

**Methods:**

This is a mixed‐methods study. We analyzed the National Family Health Survey (NFHS)‐4 data (2015–16) to estimate the prevalence and risk factors of obesity, followed by a desk review of literature and stakeholder mapping with interviews to develop policy guidance.

**Results:**

National prevalence of obesity (defined by WHO as body mass index ≥25) was comparable among pregnant (12%) and postpartum women (13%) ≥20 years of age. A high prevalence of obesity (>40%) was observed in over 30 districts in multiple states. Older maternal age, urban residence, increasing wealth quintile, and secondary education were associated with increased odds of obesity among pregnant and postpartum women; higher education increased odds among postpartum women only (OR 1.90; 95% CI, 1.44–2.52). Dietary variables were not associated with obesity. Several implementation challenges across healthcare system blocks were observed at policy level.

**Conclusion:**

Overall prevalence of obesity in India during and after pregnancy is high, with huge variation across districts. Policy and programs must be state‐specific focusing on prevention, screening, and management of obesity among pregnant and postpartum women.

## Abstract 2

Prevalence of maternal obesity in India is high with huge variations across districts, indicating the need for implementation of state‐specific policies to prevent, screen, and manage obesity as part of routine antenatal and postnatal care programs.

## 1 Introduction

Overweight and obesity, characterized by an adult body mass index (BMI, calculated as weight in kilograms divided by the square of height in meters) of 25 or more, have become a major global public health challenge with increasing rates in low‐resource countries. Between 1975 and 2016, the proportion of adult women (aged ≥20 years) with obesity increased from 6% to 15% globally.^1^, ^2^ In India, the proportion of overweight/obese adult women almost doubled from 12.6% in 2006 to 20.7% in 2016.^3^ In India, undernutrition (BMI <18.5) coexists with obesity. Women with undernutrition before and during pregnancy are at risk of giving birth to low birth weight babies, and these children are at higher risk of noncommunicable diseases (NCDs) and later obesity.^4^, ^5^, ^6^

Evidence from observational studies shows that obesity before and during pregnancy is associated with adverse health outcomes for both mothers and children, perpetuating intergenerational cycles of obesity and associated NCDs.^7^, ^8^ Obesity during pregnancy is associated with an increased risk of gestational diabetes mellitus (GDM), pre‐eclampsia, miscarriage, venous thromboembolism, infection, and hemorrhage in the mother.^9^ Furthermore, obese women may be exposed to nutrient‐poor but energy‐dense diets, contributing to adverse pregnancy outcomes.^4^ Intrauterine exposure to hyperglycemia (in GDM) and hypertension may negatively affect fetal development through epigenetic processes and lead to preterm birth, macrosomia, congenital abnormalities, stillbirth, and neonatal death.^10^, ^11^, ^12^, ^13^ Globally, maternal overweight/obesity has been reported to contribute to a 0.6 million increase in deaths and a 1.8% increase in disability‐adjusted life years between 1990 and 2010.^14^, ^15^

World Health Organization (WHO) antenatal care guidelines^16^ recommend a variety of antenatal care interventions (49 of them) of which 14 are related to nutrition, with only one having a direct bearing on obesity. Others are grouped under maternal and fetal assessments (n=13), preventive measures (n=7), common physiological symptoms (n=6), and improving health systems quality and utilization (n=9). Although there are recommendations made by the Government of India for reducing comorbidities associated with overweight and obesity, such as GDM and other NCDs, there is no guidance or standardized toolkit available on prevention, screening, and management of obesity during pregnancy. The Government of India’s *Poshan Abhiyaan* (2018–22) launched the groundbreaking mandate for convergent action across 11 government departments to ensure attainment of malnutrition‐free India and to ensure holistic development and adequate nutrition for pregnant women, mothers, and children by 2022. Currently, *Poshan Abhiyaan* targets do not include obesity in pregnancy, and existing programs focused on women in India (including antenatal care, nutrition and health education, food supplementation) have several implementation challenges.^17^ The challenges include, but are not limited to, resource constraints, gaps in the supply chain, barriers to behavior change, inadequate access to health services, policy, and governance.

Globalization and consumption of nutrient‐poor, energy‐dense processed foods and diets high in carbohydrates, coupled with the transition to sedentary occupations and reduced physical activity, have been major drivers of the global obesity epidemic in the last three to four decades.^18^, ^19^ Previous studies in India and other low‐ and middle‐income countries have consistently shown positive associations between obesity and higher socioeconomic status, higher educational qualification, and improved dietary diversity scores.^20^, ^21^, ^22^ However, there is limited evidence on risk factors for obesity in pregnancy and the postpartum period in India. Given the rising double burden of undernutrition and obesity in India, it becomes crucial to understand the prevalence of and risk factors for obesity in pregnancy and the postpartum period to develop context‐specific preventative policies.

Using data from the National Family Health Survey (NFHS)‐4^3^ and a desk review of literature with a focus on India, the aim of the present study was to examine prevalence, risk factors, and consequences of maternal obesity; and provide evidence on current policies and programs to manage maternal obesity. The hope is that findings will inform antenatal care services for prevention and management of obesity‐related risks in India. With an already strong political commitment to health and wellness through the life cycle with the launch of *Ayushman Bharat*, these findings are timely.

## 2 Materials and Methods

The geographic scope of the current study is India. The study used a mix of analytic methods including review of literature on prevalence and consequences of obesity; analyses of NFHS‐4 data (2015–16) to estimate the prevalence of and risk factors for obesity; and development of policy guidance.

### 2.1 Review of literature

We undertook a desk review of papers published in India between January 2011 and November 2019. Papers were searched in PubMed using search terms pregnan*, overweight, obes*, overnutrition, high BMI, and India. Data on prevalence, determinants, and consequences available from these papers were collated using Excel version 13 (Microsoft Corp, Redmond, WA, USA) with details of date published, authors, type of study, location, duration, and outcomes of interest.

### 2.2 Secondary analysis of NFHS‐4 (2015–16)

**Figure 1:**
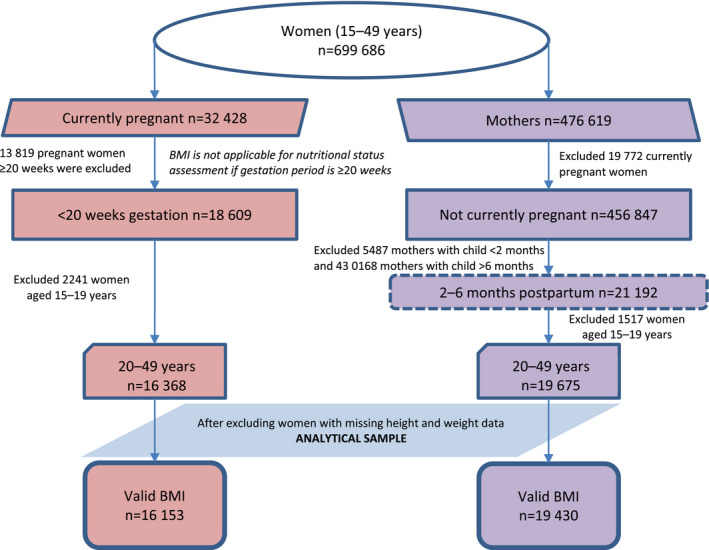
Sampling flow chart of pregnant women (<20 weeks of pregnancy) and mothers in the postpartum period (2–6 months).

Prevalence and determinants of obesity in pregnancy (<20 weeks) as well as in the postpartum period (2–6 months), were estimated using NFHS‐4 (2015–16). NFHS follows a two‐stage, stratified cluster sample design. A subsample of pregnant women (n=16 153, 20–49 years) and postpartum women (n=19 430, 20–49 years) was extracted from the NFHS‐4 sample of 699 686 women of reproductive age (15–49 years) (Fig. 1). Exclusion criteria for the sample of pregnant women included: (1) women at ≥20 weeks of pregnancy to avoid misclassification based on BMI cut‐offs or BMI for age z score; and (2) women whose height and weight measurements were not available for calculating BMI. Among postpartum women, those less than 2 months postpartum were excluded to avoid the effects of retaining weight after pregnancy, and those more than 6 months postpartum were also excluded. Due to the limited sample size, separate analyses for adolescents (15–19 years) were not undertaken in the current study.

#### 2.2.1 Variables

BMI cut‐offs for overweight/obese (≥23) and obese (≥25) among Asians,^23^, ^24^ were used to estimate prevalence among adult pregnant and postpartum women. Based on existing literature, independent variables consisted of five main groups: (1) individual characteristics; (2) household characteristics; (3) exclusive breastfeeding for postpartum women; (4) dietary intake; and (5) use of maternal health services. Individual characteristics included maternal age (defined in 5‐year age groups: 20–24, 25–29, 30–34, ≥35) age at marriage less than 18 years, parity, and education (categorized as none, primary, secondary, and higher). Household characteristics included caste, access to improved drinking water, access to improved toilets, and classification based on wealth quintiles. Dietary variables included weekly consumption of fried food and aerated drinks as indicated in the NFHS‐4 questionnaire, and daily consumption of a combination of milk/curd, pulses/beans, egg/fish/meat, and dark green leafy vegetables. Additionally, for postpartum women who used antenatal care services, the following variables were also included: consumption of 100 mg iron–folic acid tablets, deworming, receipt of supplementary nutrition, and health and nutrition education from *Anganwadi* centers. These centers, under the umbrella of the Integrated Child Development Services Scheme in India, provide a package of six services: supplementary nutrition, preschool nonformal education, nutrition and health education, immunization, health check‐up, and referral services to children aged 0–6 years and pregnant and postpartum women.^25^

#### 2.2.2 Statistical analysis

National level sampling weights were used during analysis to maximize the representativeness of the study population. Descriptive analyses were conducted to present characteristics of the study sample for women in pregnancy and the postpartum period. We developed maps depicting district‐wise cases to study the variability in the prevalence of obesity at the state level. Multivariable logistic regression was used to examine associations between obesity and its correlates. For pregnant women, the models were additionally controlled for gestational age. All data were analyzed using Stata version 15.1 (StataCorp LLC, College Station, TX, USA). *P*<0.05 was considered statistically significant.

### 2.3 Development of guidelines review grid and discussions with policymakers and implementers

A guideline review grid consisting of 10 blocks was constructed covering public health dimensions covered by the WHO and others. The blocks were: availability and accountability for guidelines; plans and financing; demand creation; leadership and governance; partnerships; information systems/monitoring and evaluation; capacity building; supply; institutionalized mechanisms research; and policy dialogue. A database of stakeholders was developed to map researchers/agencies engaged in the management of obesity using a contact list of experts from the National Centre of Excellence and Advanced Research on Diets (NCEARD), Lady Irwin College, New Delhi. A desk review of the literature was also conducted. Published and unpublished work from around 100 active stakeholders was appended to the database created through online searches.

## 3 Results

In the last decade, several researchers have investigated the prevalence of overweight and obesity and its consequences among pregnant women in community and facility settings in India, but only one study investigated the determinants.^26^, ^27^, ^28^, ^29^, ^30^, ^31^, ^32^, ^33^, ^34^, ^35^ The study cohorts spanned 10 states of India. Definition of obesity varied in the studies included (BMI ≥25 or BMI ≥30) and the reported prevalence of maternal obesity ranged from 1.1% to 46.9% (2011 to 2019). Among the consequences, overweight/obesity among pregnant and postpartum women was associated with low birth weight,^26^, ^29^ intrauterine growth restriction,^29^ macrosomia,^28^, ^31^ and adverse pregnancy outcomes such as prolonged/induced labor,^27^, ^35^ postpartum hemorrhage,^31^, ^35^ high gestational weight gain,^28^ pregnancy‐induced hypertension/pre‐eclampsia,^27^, ^29^, ^31^, ^32^, ^33^, ^34^, ^35^ and GDM.^29^, ^31^, ^32^, ^33^, ^35^

### 3.1 Findings from the NFHS‐4 analysis

#### 3.1.1 Study sample characteristics

Characteristics of the study sample are summarized in Table 1. The majority of women (~70%) were rural residents, aged 20–24 years (~50%). Around one‐third of the population belonged to socially disadvantaged groups (scheduled caste, scheduled tribe, other backward classes as defined in NFHS‐4). The majority of women (~80%) had access to improved drinking water, but less than half had access to sanitation facilities.

**Table 1:** Sociodemographic characteristics of pregnant women (<20 weeks of pregnancy) and women in the postpartum period (2–6 months), India (NFHS 4, 2015–16).

Characteristics	Pregnant women (n=16 153)	Postpartum women (n=19 430)
	No. (%)	No. (%)
Age, y		
20–24	7847 (50.6)	8774 (47.8)
25–29	5486 (34.3)	6829 (35.1)
30–34	2037 (11.3)	2651 (12.3)
≥35	783 (3.8)	1176 (4.9)
Age at marriage less than 18 y	4605 (29.3)	6605 (36.2)
Gestational age, wk		
0–4	1134 (6.3)	NA
5–8	3199 (19.4)	NA
9–12	3964 (25.5)	NA
13–16	3918 (24.2)	NA
17–20	3938 (24.6)	NA
Parity		
0	5415 (34.6)	
1	5402 (34.0)	6464 (33.8)
2	2869 (17.3)	6532 (35.3)
≥3	2467 (14.2)	6434 (30.9)
Highest educational level		
No education	4118 (25.3)	5332 (26.6)
Primary	2105 (12.1)	2709 (13.3)
Secondary	7671 (46.4)	9111 (46.4)
Higher	2259 (16.2)	2278 (13.6)
Place of residence		
Rural	12067 (70.0)	14949 (73.0)
Urban	4086 (30.0)	4481 (27.1)
Caste or tribe of the household		
Scheduled caste	3095 (21.4)	3644 (21.2)
Scheduled tribe	3040 (9.2)	4064 (11.0)
Other backward classes	6412 (45.4)	7534 (44.3)
Others	3606 (23.9)	4188 (23.4)
Improved source of drinking water	13344 (83.5)	15595 (80.9)
Improved toilet facilities	6813 (40.4)	7506 (37.0)
Wealth index		
Poorest	3693 (22.4)	4916 (24.5)
Poorer	3562 (20.2)	4560 (21.7)
Middle	3233 (20.0)	3838 (19.3)
Richer	2871 (18.2)	3253 (18.6)
Richest	2794 (19.2)	2863 (15.9)
Exclusive breastfeeding^a^	NA	NA
Dietary intake		
Consume daily milk/curd and pulses/beans or egg/fish/meat and dark green leafy vegetables	2516 (18.1)	3053 (18.0)
Eats fried food weekly	5537 (35.9)	6329 (35.2)
Consumes aerated drinks weekly	3047 (19.5)	3064 (16.4)
Maternal health services		
Consumed 100 mg iron‐folic acid tablets	NA	
Took drugs for intestinal worm	NA	
Received supplementary nutrition from AWC	NA	
Received health and nutrition education at AWC	NA	

Abbreviations: AWC, *Anganwadi* centre; NA, not applicable.

^a^ Postpartum women practicing exclusive breastfeeding (give only breast milk during the previous day).

#### 3.1.2 Prevalence of overweight and obesity

**Figure 2:**
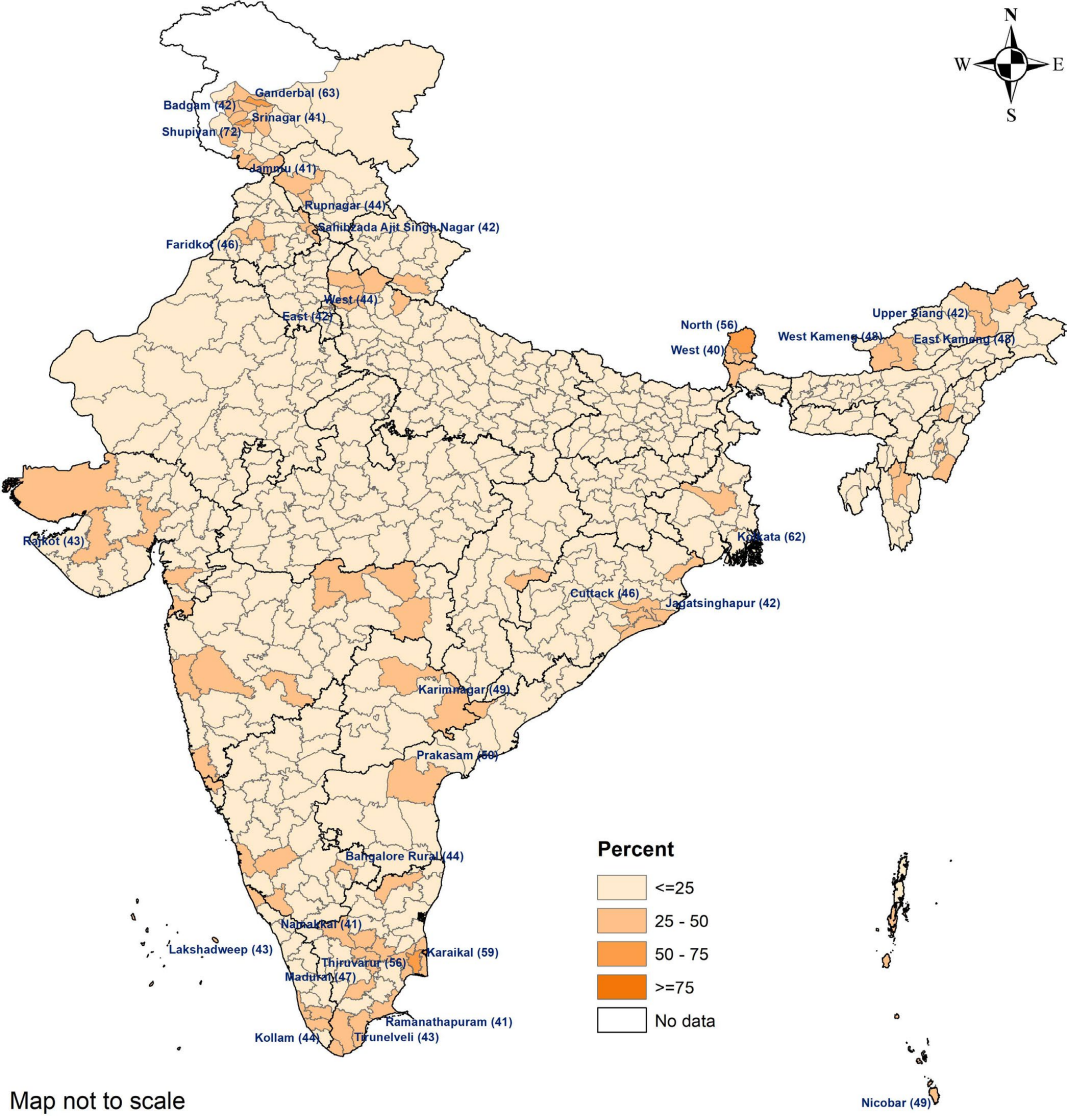
District wise prevalence of obesity (BMI ≥25) among pregnant women (<20 weeks) aged 20–49 years, India, NFHS‐4 (2015–16). Names of districts given where prevalence of obesity is greater than 40% (BMI calculated as weight in kilograms divided by the square of height in meters).

**Figure 3:**
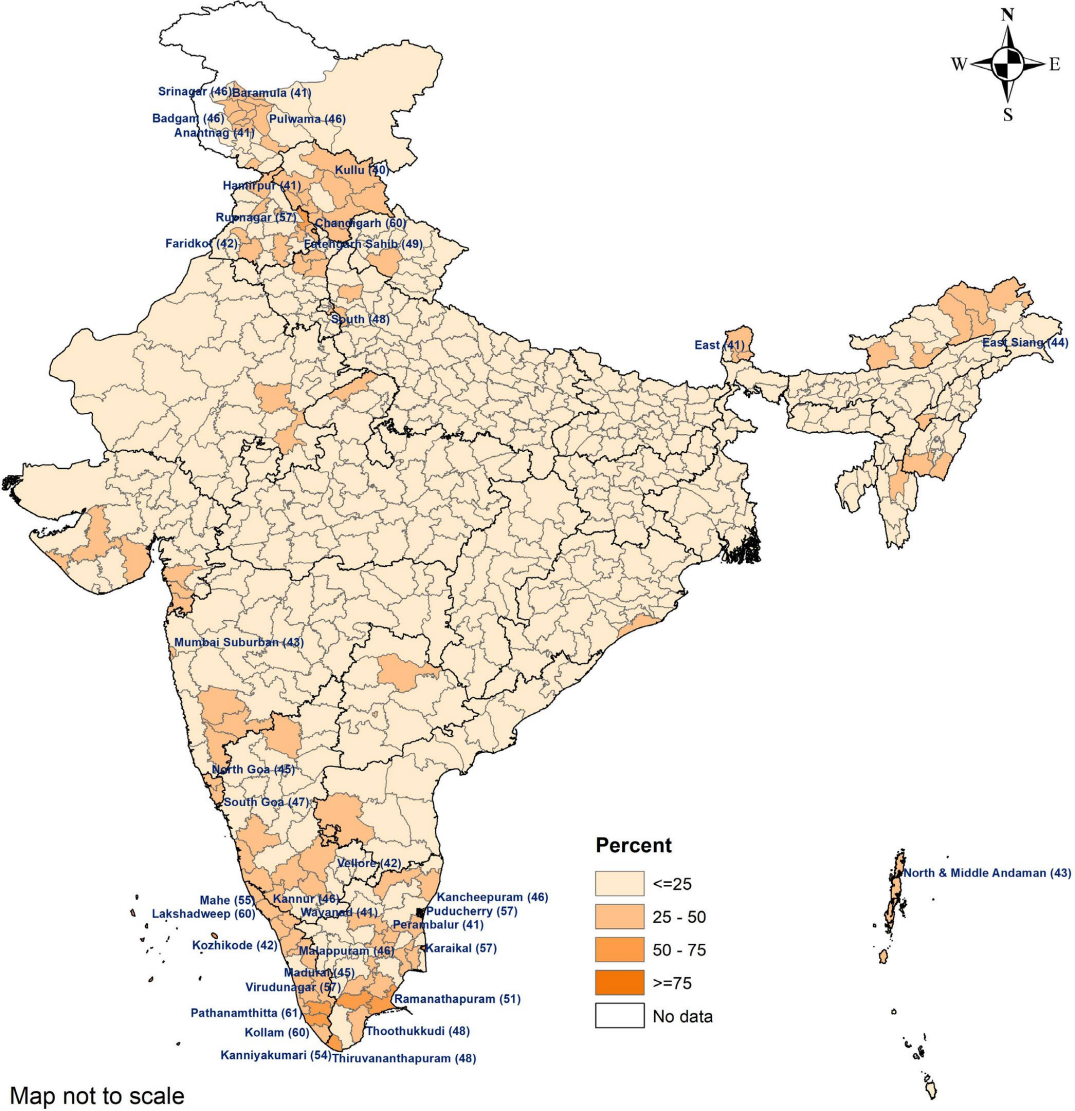
District‐wise prevalence of obesity (BMI ≥25) in postpartum women (2–6 months) aged 20–49 years, India, NFHS‐4 (2015–16). Names of districts given where prevalence of obesity is greater than 40% (BMI calculated as weight in kilograms divided by the square of height in meters).

The national prevalence of obesity was comparable in pregnancy (12%; 95% CI, 11.6–13.3) and in the postpartum period (13%; 95% CI, 12.4–13.8). Among pregnant women, the prevalence of obesity was over 40% in 31 districts, with the highest prevalence of 72% in Shupiyan district (Jammu and Kashmir) (Fig. 2). The prevalence of obesity among postpartum women was over 40% in 37 districts, with the highest prevalence of 61% in Pathanamthitta district (Kerala) (Fig. 3).

#### 3.1.3 Risk factors associated with obesity

The results from multivariate models of risk factors for obesity among women in pregnancy and the postpartum period are shown in Table 2. Older maternal age (OR 6.22; 95% CI, 4.45–8.69 and OR 4.13; 95% CI, 3.01–5.66) and increasing wealth quintile (OR 6.37; 95% CI, 4.28–9.48 and OR 8.25; 95% CI, 5.89–11.56) were significantly associated with higher odds of obesity in both pregnancy and postpartum (2–6 months). Compared to those with no education, secondary education was significantly associated with increased odds of obesity in pregnant women (OR 1.36, 95% CI, 1.05–1.75) and postpartum women (OR 1.49; 95% CI, 1.20–1.85). In contrast, higher education was significantly associated with increased odds only in postpartum women (OR 1.90; 95% CI, 1.44–2.52). Additionally, among pregnant women, the likelihood of being obese was 43% higher among urban residents (*P*<0.001).

**Table 2:** Adjusted odds ratios of obesity (BMI ≥25) among pregnant women (<20 weeks of pregnancy) and women in the postpartum period (2–6 months), India (NFHS 4, 2015–16).

Characteristics	Pregnant women (n=11 125)	Postpartum women (n=13 232)
	OR 95%CI	OR 95%CI
Age, y		
20–24	1 [Ref.]	1 [Ref.]
25–29	1.91^a^ [1.573–2.318]	1.44^a^ [1.211–1.717]
30–34	3.67^a^ [2.83–4.754]	2.71^a^ [2.166–3.403]
≥35	6.22^a^ [4.447–8.689]	4.13^a^ [3.009–5.656]
Age at marriage less than 18 y	1.19 [0.964–1.479]	1.00 [0.843–1.193]
Gestational age, wk		
0–4	1 [Ref.]	NA
5–8	0.81 [0.588–1.112]	NA
9–12	0.66^b^ [0.48–0.898]	NA
13–16	0.65^b^ [0.479–0.889]	NA
17–20	0.93 [0.685–1.25]	NA
Parity		
0	1 [Ref.]	NA
1	1.17 [0.956–1.441]	1 [Ref.]
2	1.00 [0.763–1.307]	1.12 [0.932–1.341]
≥3	1.08 [0.766–1.525]	1.21 [0.961–1.533]
Highest educational level		
No education	1 [Ref.]	1 [Ref.]
Primary	1.11 [0.834–1.484]	0.96 [0.719–1.291]
Secondary	1.36^c^ [1.054–1.747]	1.49^a^ [1.199–1.847]
Higher	1.32 [0.971–1.803]	1.90^a^ [1.439–2.517]
Place of residence		
Rural	1 [Ref.]	1 [Ref.]
Urban	1.43^a^ [1.183–1.72]	1.14 [0.969–1.347]
Caste or tribe of the household		
Scheduled caste	1 [Ref.]	1 [Ref.]
Scheduled tribe	0.70 [0.476–1.038]	0.64^b^ [0.471–0.865]
Other backward classes	0.98 [0.782–1.218]	1.19 [0.986–1.446]
Others	1.17 [0.917–1.492]	1.45^b^ [1.167–1.801]
Improved source of drinking water	0.82 [0.653–1.028]	0.91 [0.75–1.094]
Improved toilet facilities	1.07 [0.881–1.305]	0.84 [0.705–1.003]
Wealth index		
Poorest	1 [Ref.]	1 [Ref.]
Poorer	2.13^a^ [1.544–2.946]	2.28^a^ [1.727–3.022]
Middle	3.89^a^ [2.805–5.393]	3.78^a^ [2.815–5.08]
Richer	5.68^a^ [3.951–8.176]	5.21^a^ [3.804–7.132]
Richest	6.37^a^ [4.284–9.480]	8.25^a^ [5.891–11.556]
Exclusive breastfeeding^d^		0.90 [0.75–1.08]
Dietary intake		
Consume daily milk/curd and pulses/beans or egg/fish/meat and dark green leafy vegetables	1.11 [0.912–1.34]	1.04 [0.87–1.237]
Eats fried food weekly	1.11 [0.942–1.301]	1.02 [0.877–1.185]
Consumes aerated drinks weekly	0.92 [0.754–1.119]	0.97 [0.814–1.164]
Maternal health services		
Consumed 100 mg iron–folic acid tablets	NA	1.24^b^ [1.066–1.454]
Took drugs for intestinal worm	NA	1.12 [0.935–1.345]
Received supplementary food from AWC	NA	0.83^a^ [0.705–0.965]
Received health and nutrition education at AWC	NA	0.9 6[0.815–1.141]
Constant	0.03^a^ [0.02–0.055]	0.03^a^ [0.021–0.047]
Pseudo R square	0.1335	0.1455

Abbreviations: AWC, *Anganwadi* center; BMI, body mass index (calculated as weight in kilograms divided by the square of height in meters); NA, not applicable; OR, odds ratio; Ref, reference category.

^a^ *P*<0.001.

^b^ *P*<0.01.

^c^ *P*<0.05.

^d^ Postpartum women practicing exclusive breastfeeding (give only breast milk during the previous day).

Among postpartum women, those from a scheduled tribe were 46% less likely to be obese compared to those belonging to a scheduled caste (both socially disadvantaged groups), while those from other ethnic categories were more likely to be obese (OR 1.45; 95% CI, 1.17–1.80). Postpartum women consuming 100 mg iron‐folic acid tablets were 24% more likely to be obese compared to women consuming less than 100 mg iron‐folic acid tablets (*P*<0.01). Receipt of supplementary food from *Anganwadi* centers was associated with reduced odds of obesity (OR 0.83; 95% CI, 0.71–0.10). Dietary variables (weekly consumption of fried food and aerated drinks and daily consumption of a combination of milk/curd, pulses/beans, egg/fish/meat, and dark green leafy vegetable) were not associated with the odds of obesity in pregnant and postpartum women.

### 3.2 Current policy, financial, and programmatic gaps to prevent, screen, and manage maternal obesity

The Government of India’s *Poshan Abhiyaan* (2018–2022) advocates for intersectoral collaboration, behavior change communication, and counselling focused on health and nutrition services for children, pregnant, and postpartum women. In India, reduction of maternal obesity is not a prominent attribute in any national policy targets.

A standard nutrition service package has been submitted to the Ministry of Health and Family Welfare on integrating nutrition in routine antenatal care services in India at facility and community level. The service package includes five actions: nutritional assessment, classification as per nutritional risk, supplementation, referral/management, and counselling. Specific recommendations for managing maternal obesity include^36^: (1) 10 minutes’ individualized counselling to obese pregnant women by a healthcare worker on consequences of obesity during pregnancy, do’s and don’ts related to diet and exercise (avoidance of calorie‐dense, nutrient‐poor foods, maintenance of a regular meal pattern and planning meals, consumption of a balanced and diverse diet and staying hydrated, consumption of local, seasonal fruits and vegetables and home‐cooked foods, choosing smaller portions and lifestyle modifications including daily exercise for 30 minutes, adequate rest (8 hours of sleep and 2 hours of rest), avoidance of tobacco, alcohol, and other harmful substances); (2) regular home visits to assess compliance with antenatal care services (health and nutrition screening and assessment, micronutrient supplementation and deworming, targeted counselling, management and referral); (3) information on inclusion of one healthy main meal (350 Kcal) and two healthy snacks (200 Kcal each) along with two main meals (500 Kcal each) daily. The nutritional breakdown of each meal is calculated with the aim of producing a calorie deficit of 500 Kcal from the recommended dietary allowance of 2250 Kcal/d for pregnant Indian women translating into a requirement of 1750 Kcal for obese pregnant women.^37^

This service package has been tested in eight states/union territories (Bihar, Delhi, Haryana, Jharkhand, Karnataka, Madhya Pradesh, Telangana, Uttar Pradesh) in the year 2018–2019. Implementation of the maternal nutrition guidelines and toolkit in four states across all tiers of the health system at facility and community level (Bihar, Delhi, Jharkhand, Madhya Pradesh) has been undertaken with support of state health departments. However, practical experience and perspectives from policymakers and implementers suggest that there are several challenges in implementing these actions in facility or community settings (supporting information S1). Some of the challenges are lack of gestational weight gain charts and corresponding optimal weight gain recommendations for pregnant women; nutrition norms for preventing and managing maternal malnutrition lack penetration in service delivery system; budget for implementation under several heads (capacity building, equipment, supplies, human resource, dissemination, etc) not costed; engagement of private sector for demand creation; targets for maternal obesity missed in the *Poshan Abhiyaan*; and indicators for tracking and review missing from the government’s health information management system.

## 4 Discussion

This paper examined the prevalence, consequences, and risk factors of obesity in pregnant and postpartum women in India. More than 30 districts had a high prevalence of obesity (>40%) among pregnant and postpartum women, across different states. Our findings are consistent with previous estimates from Global Burden of Disease studies, which demonstrate an increasing trend in the prevalence of NCDs such as diabetes in the states of Tamil Nadu and Kerala where the prevalence of undernutrition is low, highlighting the need to focus on both sides of the spectrums of malnutrition.^5^, ^6^ Several factors were associated with increased risk of obesity that included older maternal age, urban residence, higher education, and higher socioeconomic status. This is consistent with findings from previous studies.^20^, ^38^, ^39^, ^40^

The strongest and most consistent association was observed with the wealth index for pregnant and postpartum women, indicating that dietary and lifestyle behaviors linked to obesity may be more related to increasing socioeconomic status or better standard of living than educational attainment or belonging to socially disadvantaged groups. Urban residence carried a higher risk of obesity, perhaps as a result of a sedentary lifestyle, lack of physical activity, and consumption of ultra‐processed foods. However, a recent study by Luhar et al.^38^ demonstrated that the prevalence of overweight and obesity increased in both urban and rural areas in India in the past decade (2005–2016). Given the context of demographic and nutritional transition in India, efforts to tackle the burden of obesity should, therefore, be inclusive of both urban and rural populations, prioritizing urban areas. Dietary variables did not emerge as influential factors in the regression models, although over one‐third of pregnant and postpartum women reported consuming fried foods weekly. Program factors like consumption of iron‐folic acid tablets increased the odds of obesity, and receipt of supplementary food reduced the odds of obesity; however, this is likely attributable to poor accuracy in self‐reported measures^41^ and selection bias in program use.

The current study has several shortcomings. No information on physical activity was available, and data from dietary recall were limited. Data on consumption of sugar‐sweetened beverages, the quantity of processed foods and carbohydrates, along with physical activity levels would be important in understanding the association of obesity with the predictors discussed above.

Given the high prevalence of maternal obesity, we recommend the following. Firstly, advocating with the Ministry of Health and Family Welfare, Government of India for the inclusion of BMI‐based screening for overweight/obesity (BMI ≥23) and obesity (BMI ≥25) for all pregnant women contacted within the first trimester by a healthcare service provider, and weight gain over 3 kg per month after the first trimester as a high‐risk indicator. Secondly, a closer review of maternal nutrition services in districts with a high prevalence of obesity is needed. Existing partnerships with other departments such as the Ministry of Women and Child Development and National/State Rural Livelihood Mission through their Self‐Help Group platforms, *Panchayati Raj* Institutions etc. to provide rations/supplementary foods and use behavior change communication for better counselling in these areas should be strengthened. The states of Bihar, Delhi, Jharkhand, and Madhya Pradesh may be prioritized as they are already implementing the five actions maternal nutrition algorithm and communication toolkit. Thirdly, innovative strategies should be developed to counsel pregnant women and demonstrate low‐cost, nutrient‐dense meals to manage weight gain and follow up pregnant women to understand the effect of counselling on weight maintenance. The FIGO (International Federation of Gynecology and Obstetrics) Nutrition Checklist should also be used by healthcare practitioners for dietary recommendations while consulting women in early pregnancy.^4^ Furthermore, healthcare practitioners should consider, where feasible, screening and counselling for obesity during pregnancy and postpartum, and achieving a healthy weight before pregnancy. Fourthly, a sample budget with actual denominators from a state using a Government of India template should be created to serve as a ready reckoner to ensure that sufficient resources are allocated in planning for implementation of the guidelines and toolkit across the country. Lastly, to further intensify the efforts, partnerships need to be formed with research groups working on pregnancy or preconception cohorts to develop India‐specific gestational weight gain charts as per prepregnancy BMI and with a focus on improving health, dietary, and lifestyle behaviors among adolescents as the younger age of marriage in India is considerably high.

In conclusion, our results indicate a comparable national prevalence of obesity among pregnant (12%) and postpartum women (13%) with a higher prevalence of obesity (>40%) in more than 30 districts scattered across the country. Older maternal age, urban residence, higher education, and higher socioeconomic status increased the likelihood of maternal obesity. With ongoing demographic and nutritional transitions in India, policies and programs must be state‐specific, consider the cultural and social influences on dietary patterns, and focus on prevention, screening, and management of obesity in pregnancy and the postpartum period. These must be established as a priority to reduce the burden of morbidity and mortality for both mothers and their infants.

## Author Contributions

MC, VS, MS, and AB conceptualized the paper and drafted it with contributions from NK, DB, and MAH. KDS conducted the statistical analysis. All other authors reviewed the manuscript and contributed to the interpretation of findings. All authors agreed to the final version of the paper.

## Disclaimer

This is a working document. It has been prepared to facilitate the exchange of knowledge and to stimulate discussion. The statements in this publication are the views of the author(s) and do not necessarily reflect the policies or the views of UNICEF. The designations employed in this publication and the presentation of the material do not imply on the part of UNICEF the expression of any opinion whatsoever concerning the legal status of any country or area, or of its authorities or the delimitations of its frontiers. The text has not been edited or fact‐checked to official publications standards and UNICEF accepts no responsibility for error.

## Conflicts of Interest

The authors have no conflict of interest.

## Supporting information


**Supporting information S1**. Current status and gaps in implementing evidence‐based, consensus‐driven five actions for maternal obesity at facility and community level.
