# Supplementary material for: Population estimates, consequences, and risk factors of obesity among pregnant and postpartum women in India: Results from a national survey and policy recommendations
Source: Int J Gynaecol Obstet. 2020 Sep 7;151(Suppl 1):57–67. doi: 10.1002/ijgo.13319 (PMC7590096; doi:10.1002/ijgo.13319)
Supplement: Supplementary file 1 — Supporting information S1. Current status and gaps in implementing evidence‐based, consensus‐driven five actions for maternal obesity at facility and community level. [file IJGO-151-57-s001.doc]

**Supporting information S1.** Current status and gaps in implementing evidence-based, consensus-driven five actions for maternal obesity at facility and community level.

| **Pillars** | **Status** | **Gaps** |
| --- | --- | --- |
| 1. Implementation guidelines: availability and accountability | - Maternal nutrition (MN) guidelines tested and available for rollout.   Resources available include:  Algorithm (5 actions) for facility and community level  Flipbook for group counselling  By-gestational month cards for group counselling  At-risk cards/condition-specific cards for individual counselling  Recipe book on 9 conditions  Body Mass Index (BMI) chart for field workers  Computer-assisted personal interviewing (CAPI) based tool on CSPro for data entry  The MN guidelines are being implemented at: Bihar, Delhi, Madhya Pradesh, Jharkhand   - Specific components of these guidelines are already included in national programs   *Surakshit Matritva Aashwasan* (SUMAN), Ministry of Health and Family Welfare: Recipe book on 9 conditions, *Thali* models (normal weight, thin, obese, and anemic), Leaflet for pregnant women (normal weight, thin, obese, and anemic), by-gestational month cards, flipbook  Ministry of Woman and Child Development: Regional recipes leaflet for pregnant women (normal weight, thin, obese, and anemic) [for North, North-East, Central, West and South regions] | - Lack of gestational weight gain charts and corresponding optimal weight gain. Standard of 10–12 kg being followed without base weight reference point - Nutrition assessments and actions undertaken by Auxiliary Nurse Midwifes (ANM), Medical Officers (MOs) and specialists but neither consistently nor comprehensively done (weight measurement and hemoglobin assessment most frequently done) |
|  | - Micronutrients covered under guidelines: Iron, Folic acid, Calcium, Iodine, Vitamin D | - Emerging micronutrient deficiencies: Vitamin B6, B12, and Zinc unaddressed in draft guidelines |
|  | - Guidelines Gestational Diabetes Mellitus, Tuberculosis, Human Immunodeficiency Virus have nutrition component | - Guidelines on thyroid disorders, which are now commonly reported in pregnancy (and other medical conditions), required to conduct universal testing, especially where the problem is of public health significance. A nutrition management section is needed |
|  | - Roles and accountability for preventing and managing maternal malnutrition defined in draft guidelines (Facility: ANM, Community Health Officer (CHO) and MO; Community: ANM, Accredited Social Health Activist (ASHA) and *Anganwadi* worker (AWW)) | - Available nutrition norms need to penetrate service delivery system (increase provider awareness) |
| 2. Plans and financing | - Head available under National Health Mission:   Procurement (iron-folic acid, calcium, albendazole, iron sucrose and hemoglobinometer)  ASHA Incentive (mobilizing lactating women under *Anemia Mukt Bharat*)  Capacity Building under Maternal Health  Strengthening maternal health service (community based, facility based, *Janani Shishu Suraksha Karyakram* (JSSK) Diet Norms etc)  Information Education Communication (IEC)/Social Behavior Change Communication (SBCC)  Printing  Flexibility under each head | - Heads available but budget amounts need to match requirements for training, outreach, equipment, and supplies. The gaps vary by state - Capacity building (maternal nutrition trainings with estimated cost needs to be incorporated within the available budget heads) - Maternal Nutrition IEC/SBCC and printing activities need to be listed and costed |
| 3. Demand creation | - Standard communication/counselling materials for pregnancy nutrition, nutritional disorders and 9 medical co-morbidities available but yet to be implemented across the country | - Stakeholder consensus on consistency in using messages across regions and platforms (with localization as needed) - Engagement of private sector providers in demand creation |
| 4. Leadership and governance | - *POSHAN Abhiyaan* targets being closely monitored | - Overweight/obesity through life course (or at any specific stage in life course) is missed in the *Poshan Abhiyaan* targets - Management strategies for obese women require coordination across woman and child development, rural development, and other departments - Review of maternal nutrition initiatives within *Poshan Abhiyaan* |
| 5. Partnerships | - Maternal nutrition consortium created - National Centre of Excellence and Advanced Research on Diets (NCEARD), New Delhi, India established to forge national and regional partnerships | - Models for engaging private sector providers needed - Regional centers of excellence on maternal nutrition needed through government or corporate funding support |
| 6. Information systems/monitoring and evaluation | - The Reproductive and Child Health portal and Health Management Information System (HMIS) combined covers monitoring targets for pregnancy anemia (process and outcome level)   Maternal nutrition scorecards based on HMIS Indicators have been developed | - Indicators for BMI-based tracking and counselling services needed |
| 7. Capacity building | - Training resources are available and implemented in 4 states (Bihar, Delhi, Jharkhand, Madhya Pradesh) - Master trainers:   Madhya Pradesh: 410  Bihar: 40  Jharkhand: 187  Delhi: 137 | - Identify platforms where half-day trainings on maternal nutrition for ASHA, AWW, ANM, MO, CHO can be integrated (state level) |
| 8. Supply | - Equipment available: digital weighing scale - Medicines/supplements: Folic acid tablets, iron-folic acid, iron sucrose, calcium tablets, albendazole, glucose load | - Equipment needed: stadiometer, non-stretchable inelastic tape, hemoglobinometer - Procurement challenges for medicines/supplements |
| 9. Institutional mechanism | - NCEARD technical support unit for maternal nutrition | - Regional centers required especially where maternal obesity prevalence is >40% |
| 10. Research and policy dialogue | - Consultation meetings (13) to develop the maternal nutrition guidelines, algorithms and communication materials - Maternal Nutrition symposium series planned with themes: severe thinness, obesity, anemia, and depression - Results on management of severely thin pregnant women and mothers at Nutrition Rehabilitation Centers | - Gestational weight gain monitoring charts for normal, thin, and obese pregnant women - Managing gestational weight in thin and obese mothers - Operational models where thinness and obesity are managed through health systems (engaging health and wellness centers) - Multiple micronutrient supplements vs iron-folic acid in pregnant women |
